# Ewing Sarcoma: Rare Metastasis to the Pancreas

**DOI:** 10.14309/crj.0000000000000930

**Published:** 2022-12-26

**Authors:** Bhavna A. Guduguntla, Jiaqi Shi, Richard S. Kwon

**Affiliations:** 1 University of Michigan Medical School, Ann Arbor, MI; 2 Department of Pathology and Clinical Labs, University of Michigan, Ann Arbor, MI; 3 Division of Gastroenterology and Hepatology, Department of Internal Medicine, University of Michigan, Ann Arbor, MI

## Abstract

Ewing sarcoma is a highly aggressive malignancy of bone or soft tissue, which may present with metastasis for 20%–25% of patients. The most common sites of metastatic lesions are the bone, bone marrow, and lungs. When metastatic lesions present within rare visceral sites, such as the pancreas, it may lead to an incorrect diagnosis of small cell neuroendocrine carcinoma. We report a case of a 37-year-old man with metastatic Ewing sarcoma involving the pancreas, confirmed by imaging, sequencing, fluorescence in situ hybridization, and histology, which was initially mistaken for small cell neuroendocrine carcinoma.

## INTRODUCTION

Ewing sarcoma (ES) is a highly aggressive malignancy of bone or soft tissue that may initially present with metastasis in approximately 20%–25% of cases.^1^ It most commonly affects children and adolescents younger than 20 years.^2,3^ Extraosseous ES primarily originates in the cervical muscles, pleural cavities, thoracic wall, and gluteal muscle. The most common locations of metastasis are the lungs, bone, and bone marrow, whereas other sites are exceedingly rare.^1^ Primary tumors or metastatic lesions in rare sites may be mistaken as a neuroendocrine carcinoma. Differentiating between these diagnoses is critical in optimizing therapeutic regimens and patient outcomes.^4^ We present a case of ES adjacent to the prostate within the pelvic cavity with metastases to the pancreas that was initially diagnosed as a poorly differentiated neuroendocrine carcinoma. There have been 5 documented cases of metastatic ES involving the pancreas.^5–8^

## CASE REPORT

**Figure 1.**
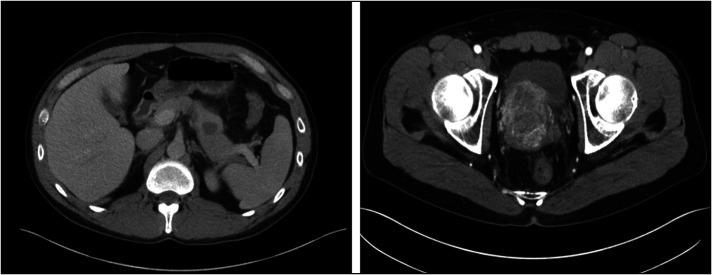
Abdominal and pelvic computed tomography showing a 4 cm by 5.1 cm pelvic mass protruding into the bladder (right) and a 2.2 cm hypodense lesion within the pancreatic body (left).

**Figure 2.**
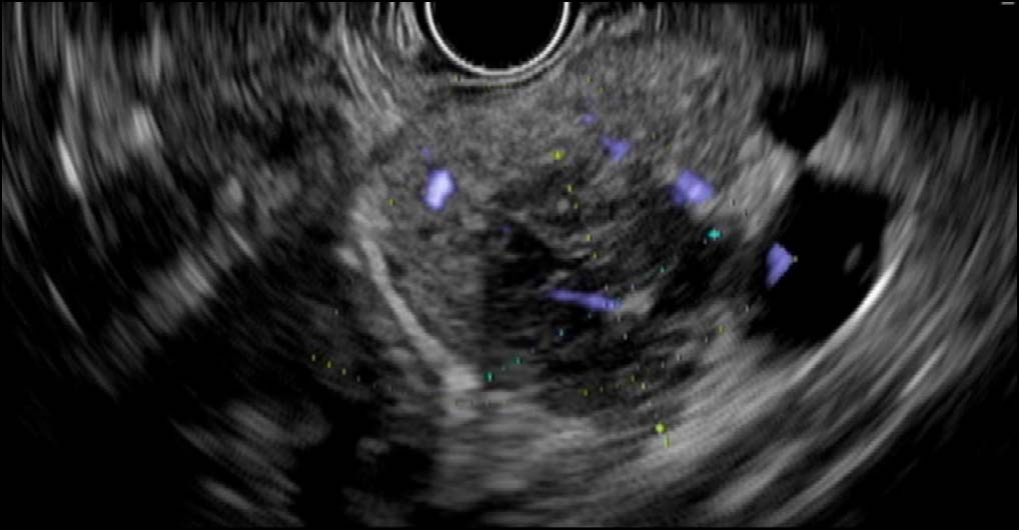
Endoscopic ultrasound demonstrating a 2.8 cm by 2.7 cm heterogeneous mass with irregular margins in the pancreatic body.

**Figure 3.**
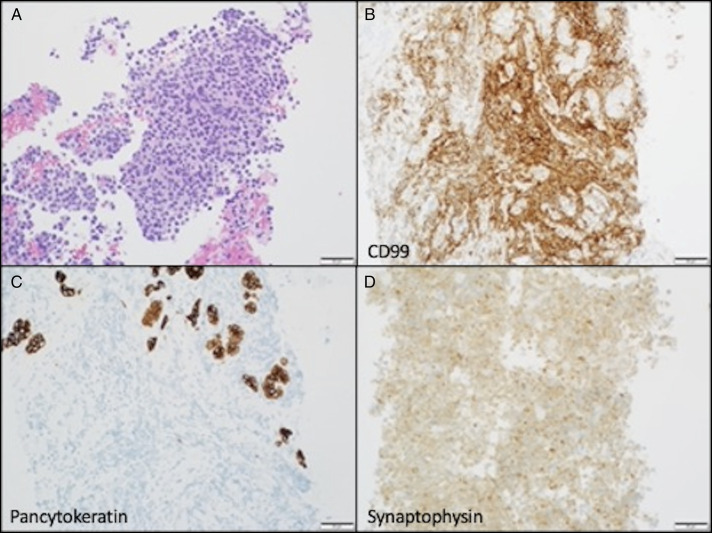
Ewing sarcoma in the pancreas. (A) Representative hematoxylin and eosin staining of the pancreatic mass biopsy. Immunohistochemistry staining of the pancreatic mass biopsy for CD99 (B), pancytokeratin (C), and synaptophysin (D). Scale bar = 50 μm.

A 37-year-old man presented with acute-onset nausea, vomiting, and epigastric pain, along with 3 weeks of progressive urinary urgency, polyuria, and dysuria. Initial laboratory values in the emergency department were notable for an abnormal lipase of 3758 U/L (normal range: 12–53 U/L) and abnormal C-reactive protein of 1.7 mg/dL (normal range: 0.0–0.6 mg/dL). His family history was significant for colon cancer (mother) and lung cancer (paternal uncle). The patient had a 20-pack-year smoking history. Abdominal and pelvic computed tomography showed a 4 cm × 5.1 cm pelvic mass protruding into the bladder and a 2.2 cm hypodense lesion within the pancreatic body (Figure 1). A transrectal biopsy of the pelvic mass was suspicious of neuroendocrine tumor. Endoscopic ultrasound demonstrated a 2.8 cm × 2.7 cm heterogeneous mass with irregular margins in the pancreatic body (Figure 2). A fine-needle core biopsy was obtained. Histologically, the pancreas mass was a poorly differentiated malignant neoplasm. The neoplastic cells had hyperchromatic and relatively small uniform nuclei with numerous mitotic figures and minimal cytoplasm (Figure 3). Immunohistochemical stains were performed, and the neoplastic cells were diffusely positive for synaptophysin, but negative for chromogranin and trypsin with a Ki-67 proliferative index of >95% (Figure 3). Based on the histology and immunoprofile of this tumor, it was diagnosed as poorly differentiated neuroendocrine carcinoma (small cell carcinoma). The patient began cisplatin and etoposide, but imaging after 2 months revealed disease progression with tumor expansion. This prompted additional cytologic analysis to clarify the diagnosis.

Next-generation sequencing revealed CCND1 overexpression, EWRS1-FLI1 chromosomal rearrangement, stable microsatellite instability, tumor mutation burden 3.7 m/MB, programmed death-ligand 1 tumor proportion score < 1, and carbamoyl phosphate synthetase 1, suggestive of ES. A diagnosis of ES was confirmed by fluorescence in situ hybridization of the pancreatic mass, which was positive for the EWSRI-FLI1 (22q12) chromosomal rearrangement. Further immunohistochemical staining demonstrated neoplastic cells diffusely positive for CD99 and negative for cytokeratin, supporting the diagnosis of ES. The patient consequently began a chemotherapy regimen of vincristine, actinomycin, and cyclophosphamide. One year later, he is undergoing concomitant radiation therapy.

## DISCUSSION

ES disproportionally affects younger individuals and is most frequently diagnosed around the age of 15 years.^1^ Less than 30% of ES cases present with metastasis at the time of diagnosis, which are often resistant to treatment.^1^ The pancreas is an exceedingly rare site of metastatic disease, especially for ES.^5–8^ Our patient presented with a rare extraosseous ES with an exceedingly rare site of metastases. Like our patient, the most common initial symptoms of primary pancreatic ES or metastatic ES with lesions to the pancreas include abdominal or epigastric pain, jaundice, nausea, and vomiting.^3,7^ His symptoms of polyuria and polydipsia were consistent with his pelvic disease.^3,7,9^

There have been 5 documented cases of metastatic ES to the pancreas, with 4 presenting as a recurrence several years after their initial diagnosis and only 1 case similar to ours, presenting as metastasis at the time of diagnosis.^5–8^ Although our case presented with metastasis to the pancreatic body, they have been reported to occur anywhere in the pancreas, including the head, neck, and tail.^5–8^ All 5 patients presented with variable sites of pain initially while 2 patients presented with jaundice.^5–8^ Two cases were diagnosed by computed tomography-guided percutaneous biopsy.^5,6^ One diagnosis was made by endoscopic ultrasound-fine-needle biopsy.^7^

ES may be mistaken for a small cell neuroendocrine carcinoma, especially when present near or within the pancreas where small cell neuroendocrine carcinoma is more typical.^4^ These 2 tumors share morphological characteristics, such as small, round cells that contain minimal cytoplasm.^1,2^ On imaging, both tumors typically have irregular borders and heterogeneous enhancement.^3^ Differentiation between these 2 diseases is critical because they require disparate treatment regimens. Another rare pancreatic finding that should remain on the differential in a young person is solid pseudopapillary neoplasms. Solid pseudopapillary neoplasms are usually well-defined, heterogeneous (cystic), benign tumors on imaging. Their diagnosis is confirmed by characteristic immunohistochemical analyses.^10^

Prior literature shows that approximately 85% of patients with ES carry at (11;22)(q24;q12) chromosomal translocation, which most commonly results in the EWSR-FLI1 fusion gene.^2^ This gene is an abnormal transcription factor that can affect prognosis with effects that vary by the specific combination of exons. Analysis by fluorescence in situ hybridization or reverse transcription polymerase chain reaction can identify the EWSR-FLI1 fusion gene, which is specific for ES.^1,2^ Immunohistochemical analysis of the tumor cells may or may not provide further clarification of the diagnosis because both ES and small cell neuroendocrine carcinoma can be positive for neuroendocrine markers, such as synaptophysin, and cytokeratin. The presence of the EWSR-FLI1 gene results in the expression of cell-surface marker CD99, which is present in approximately 95% of ES. However, because CD99 is a widely expressed glycoprotein, present in both healthy and cancerous tissue, it is nonspecific and primarily used as a supplemental marker of ES.^1^ Our patient displayed both the EWSR-FLI1 fusion gene and CD99 positivity.

In conclusion, this case of metastatic ES to the pancreas highlights the importance of considering this diagnosis within the differential for younger patients who present with a pancreatic mass, particularly if the tumor is initially diagnosed as a neuroendocrine carcinoma. ES tumors may be mistaken for small cell neuroendocrine carcinoma because of morphological and immunohistochemical similarities, which delays the administration of the correct chemotherapy regimen and may create suboptimal patient outcomes. Molecular studies are required to confirm the diagnosis.

## DISCLOSURES

Author contributions: BA Guduguntla wrote the manuscript. J. Shi provided the pathology images and revised the manuscript. RS Kwon revised the manuscript, provided the radiology images, and is the article guarantor.

Financial disclosure: J. Shi was supported in part by the National Cancer Institute of the National Institutes of Health under award number K08CA234222.

Informed consent was obtained for this case report.
